# Isolation and Characterization of a Highly Virulent Getah Virus Causing Acute Lethal Infection in Neonatal Piglets

**DOI:** 10.1155/tbed/2271732

**Published:** 2026-07-02

**Authors:** Zhengxin Yang, Weidong Yan, Xuefei Wang, Mengjia Zhang, Zongyang Peng, Mengdi Zhang, Ahmed H. Ghonaim, Xuexiang Yu, Thi Trang Vy, Xugang Ku, Mingguang Zhou, Gaoyuan Xu, Anan Jongkaewwattana, Qigai He, Wentao Li

**Affiliations:** ^1^ College of Veterinary Medicine, Huazhong Agricultural University, Wuhan, 430070, China, hzau.edu.cn; ^2^ Hainan Research Institute, Huazhong Agricultural University, Sanya, 572025, China, hzau.edu.cn; ^3^ National Key Laboratory of Agricultural Microbiology and Hubei Hongshan Laboratory, Huazhong Agricultural University, Wuhan, 430070, China, hzau.edu.cn; ^4^ Wuhan Keqian Biology Co. Ltd., Wuhan, 430072, China; ^5^ College of Informatics, Huazhong Agricultural University, Wuhan, 430070, China, hzau.edu.cn; ^6^ Virology and Cell Technology Research Team, National Center for Genetic Engineering and Biotechnology (BIOTEC), National Science and Technology Development Agency (NSTDA), Pathum Thani, 12120, Thailand, nstda.or.th; ^7^ Hubei Jiangxia Laboratory, Wuhan, 430200, China

**Keywords:** Getah virus, neonatal piglets, phylogenetic analysis, swine disease, viral virulence, virus isolation

## Abstract

Getah virus (GETV) is a mosquito‐borne alphavirus with an expanding host range and an increasing impact on swine production, particularly in neonatal piglets. In this study, a severe disease outbreak characterized by acute diarrhea and high mortality occurred among suckling piglets on a commercial pig farm in central China. Comprehensive pathogen screening excluded common bacterial pathogens and major enteric viruses, whereas GETV RNA was consistently detected in multiple tissues. A GETV strain, designated GETV‐WH, was successfully isolated from lung tissue and propagated in BHK‐21 cells, where it reproducibly induced pronounced cytopathic effects (CPEs). The isolate was confirmed by immunofluorescence assay (IFA) using a GETV E2–specific monoclonal antibody and further purified by plaque assays. Growth kinetics analysis demonstrated efficient viral replication, with peak titers exceeding 10^8^ TCID_50_/mL at 24 h postinfection. Experimental infection of 5‐day‐old piglets resulted in rapid disease progression characterized by hypothermia, systemic viral dissemination, severe multiorgan lesions, and 100% mortality within 2 days postinoculation, indicating high virulence of the isolate in neonatal piglets. Complete genome sequencing and comparative analysis identified multiple amino acid substitutions relative to the reference strain MM2021, including two unique substitutions located in NSP1 and the E2 protein. Phylogenetic analysis based on complete genome sequences placed GETV‐WH within genetic Group III, where it formed a distinct branch among contemporary Chinese strains. Collectively, these findings demonstrate that GETV‐WH is a highly pathogenic GETV strain capable of causing acute systemic infection and lethal disease in neonatal piglets, highlighting the potential threat posed by highly virulent GETV variants to swine health.

## 1. Introduction

Getah virus (GETV) is a mosquito‐borne virus belonging to the genus *Alphavirus* within the family *Togaviridae* [1]. The virus was first isolated in 1955 from Culex mosquitoes in Malaysia and has since been reported across Asia and the Pan‐Pacific region [2]. GETV exhibits a broad host range, infecting multiple animal species, including horses, pigs, cattle, and blue foxes [3, 4]. Experimental studies have further demonstrated susceptibility in rabbits, apes, chimpanzees, and guinea pigs [5], while serological surveys have detected GETV‐specific antibodies in a wide variety of domestic animals, wildlife, and humans, indicating extensive viral circulation and cross‐species transmission [6].

In recent years, GETV has been increasingly detected on pig farms, where infection is frequently associated with severe clinical disease, particularly in neonatal piglets. Affected piglets typically present with fever, diarrhea, and rapid clinical deterioration, often resulting in high mortality rates [7]. Such outbreaks have caused substantial economic losses due to increased piglet mortality, reduced production efficiency, and elevated disease control costs associated with disease control and prevention. In China, GETV infections have been reported in multiple animal hosts across 23 provinces, underscoring their widespread distribution and growing epidemiological significance [6].

Despite this broad geographic presence, swine‐derived GETV infections have not yet been well‐documented in Hubei Province. The lack of regional data has limited understanding of the local epidemiology, viral circulation, and transmission dynamics of GETV in this area. In this study, we investigated a severe disease outbreak affecting piglets in Wuhan, Hubei Province, and reported the first isolation and molecular characterization of a swine‐derived GETV strain from this region. These findings expand the known geographic range of GETV, provide new insights into its molecular evolution, and highlight the urgent need for enhanced surveillance of GETV in swine populations.

## 2. Materials and Methods

### 2.1. Case Presentation and Sample Collection

In June 2024, an acute disease outbreak occurred on a commercial pig farm in Wuhan, Hubei Province, China. The farm housed 149 sows that had been introduced at ~60 days of gestation and were routinely vaccinated against major swine viral diseases prior to farrowing. During the production cycle, ~1800 piglets were born. Piglets aged 8–24 days were identified as the affected population and were included in the etiological investigation. Samples including whole blood, lung, brain, inguinal lymph nodes, and intestinal tissues were collected from diseased piglets. All samples were processed immediately and stored at −80°C until further analysis.

### 2.2. Pathogen Detection and Immunohistochemistry (IHC)

To identify the etiological agent, systematic screening for common swine pathogens was performed. Major bacterial pathogens associated with respiratory or enteric diseases in pigs, including *Glaesserella parasuis*, *Pasteurella multocida*, *Actinobacillus pleuropneumoniae*, *Bordetella bronchiseptica*, *Streptococcus suis*, and *Salmonella* spp., were examined by PCR following bacterial isolation. Briefly, tissue samples were cultured on appropriate agar plates, representative colonies were selected, and species‐specific PCR assays were conducted for bacterial identification. Viral pathogens, including porcine epidemic diarrhea virus (PEDV), transmissible gastroenteritis virus (TGEV), and porcine rotavirus (PoRV), were detected by quantitative reverse transcription PCR (RT–qPCR) (Vazyme, China). Detection of GETV was also performed using RT–qPCR [8], and cycle threshold (Ct) values were used to estimate viral load distribution among different tissues.

For IHC analysis, lung, jejunum, and lymph node tissues were fixed in 4% paraformaldehyde, embedded in paraffin, and sectioned. Tissue sections were incubated with a laboratory‐generated monoclonal antibody against the GETV E2 protein. Immunoreactive signals were visualized to evaluate the tissue distribution of GETV antigens.

### 2.3. Virus Isolation and Immunofluorescence Assay (IFA)

For virus isolation, ~1 g of lung tissue was homogenized in 1 mL of Dulbecco’s Modified Eagle’s Medium (DMEM) and centrifuged to remove cellular debris. The supernatant was filtered through a 0.22 μm membrane and inoculated onto BHK‐21 cell monolayers. Virus isolation was performed through three consecutive blind passages, and cytopathic effects (CPEs) were monitored daily.

IFA was conducted to confirm GETV infection in cultured cells [9]. BHK‐21 cell monolayers were infected with culture supernatants from CPE‐positive cells and incubated for 24 h in DMEM supplemented with 2% fetal bovine serum (FBS). Cells were then fixed with 4% paraformaldehyde, blocked with 5% skim milk, and incubated with a laboratory‐prepared monoclonal antibody against the GETV E2 protein, followed by incubation with an Alexa Fluor 594–conjugated secondary antibody (ABclonal, China). Cell nuclei were counterstained with DAPI, and fluorescence signals were observed using a fluorescence microscope.

### 2.4. Plaque Purification Assay and Viral Growth Curve

Plaque purification was performed to obtain a clonal GETV isolate. Briefly, BHK‐21 cells were seeded in six‐well plates and infected with serial 10‐fold dilutions of virus‐containing supernatants. After adsorption for 1 h at 37°C, the inoculum was removed, and cells were overlaid with DMEM containing agar and 2% FBS. Plates were incubated at 37°C with 5% CO_2_ for 3–4 days until well‐isolated plaques were visible. Individual plaques were carefully picked and transferred into DMEM for virus amplification in BHK‐21 cells. This plaque purification procedure was repeated for three consecutive rounds to obtain a purified virus isolate.

Viral growth kinetics were determined using BHK‐21 cells. Cell monolayers cultured in six‐well plates were infected with the purified GETV isolate at a multiplicity of infection (MOI) of 0.1 and incubated at 37°C for 1 h. After adsorption, cells were washed twice with phosphate‐buffered saline (PBS) and maintained in DMEM supplemented with 2% FBS. Culture supernatants were collected at 12, 24, 36, 48, 60, and 72 h postinfection and stored at −80°C until analysis. Viral titers were determined as TCID_50_ on BHK‐21 cells and calculated using the Reed–Muench method to generate the viral growth curve.

### 2.5. Genome Sequencing and Phylogenetic Analysis

Viral RNA was extracted from the plaque‐purified GETV isolate using a commercial viral RNA extraction kit (Bioer, China) according to the manufacturer’s instructions. The complete viral genome was amplified by reverse transcription PCR using primer sets targeting overlapping genomic regions of GETV, adopted from previously published studies [10]. Reverse transcription and PCR amplification were performed using a commercial kit (Vazyme, China), following the manufacturer’s protocols. PCR products were separated by agarose gel electrophoresis, purified using a gel extraction kit (Tiangen, China), and submitted to a commercial sequencing service provider (Hzykang, China) for nanopore sequencing.

Raw sequencing data were assembled to obtain the complete genome sequence. For comparative genomic analysis, 85 complete GETV genome sequences were retrieved from the NCBI GenBank database, and the GETV‐WH strain identified in this study was included. Open reading frames (ORFs) were extracted and translated into amino acid sequences using MEGA11 software. Amino acid sequences were aligned, and pairwise comparisons were performed to identify amino acid substitutions in GETV‐WH relative to the reference strain MM2021. The frequency of each substitution among GETV strains was calculated to determine whether mutations were common, rare, or unique. Amino acid substitution frequencies and visualizations were generated using R software [11].

For phylogenetic analysis, complete genome nucleotide sequences were aligned using MEGA11 software [12]. The optimal nucleotide substitution model was determined prior to tree construction. Phylogenetic trees were constructed using the neighbor‐joining method with 1000 bootstrap replicates. Trees were visualized and annotated using ChiPlot (https://www.chiplot.online/), with host species, geographic origin, and genetic group classifications indicated [13].

### 2.6. Animal Experiments and Virus Distribution in Organs

Animal experiments were conducted to evaluate the pathogenicity and tissue distribution of the GETV‐WH strain in piglets, in accordance with institutional ethical guidelines (Approval Number SOR‐AR‐022) [14]. Ten clinically healthy 5‐day‐old piglets were randomly assigned to an experimental group and a control group. Piglets in the experimental group were intramuscularly inoculated with 2 mL of DMEM containing 2 × 10^8^ TCID_50_ of the GETV‐WH strain, while control piglets received an equivalent volume of DMEM. Clinical signs, including rectal temperature and diarrhea, were monitored and recorded daily. At 7 days postinoculation, all piglets were humanely euthanized and necropsied. Tissue specimens, including heart, lungs, liver, spleen, kidneys, lymph nodes, intestine, and brain, were collected for viral load determination and pathological examination [7]. Total RNA was extracted from tissue samples (Vazyme, China), and GETV RNA levels were quantified by real‐time RT–PCR using previously described primers.

For histopathological analysis, tissue samples were fixed in 4% paraformaldehyde, embedded in paraffin, sectioned at a thickness of 4–6 μm, and stained with hematoxylin and eosin (H&E). Histological lesions were examined under a light microscope.

### 2.7. Statistical Analysis

Statistical analyses were performed using SPSS 25.0 and GraphPad Prism 8.4.3. Differences between groups were analyzed using Student’s *t*‐test or one‐way analysis of variance (ANOVA). A two‐sided *p*‐value of <0.05 was considered statistically significant [15].

## 3. Results

### 3.1. Clinical and Pathological Findings

Disease was observed in suckling piglets aged 8–24 days, with younger piglets exhibiting more severe clinical manifestations. Affected piglets initially presented with depression, pyrexia (40.6–41.0°C), and constipation. As the disease progressed, diarrhea developed during the middle to late stages of infection and was predominantly characterized predominantly by yellow, loose feces; watery diarrhea was rarely observed. Vomiting occurred in a subset of affected animals. Death typically occurred within ~10 h following the onset of diarrhea. No obvious neurological signs were observed throughout the course of the disease. Clinically affected piglets exhibited visible cyanosis of the abdominal, submandibular, and perioral skin, which blanched upon digital pressure. The overall morbidity exceeded 40%, and the mortality rate was greater than 50%.

**Figure 1:**
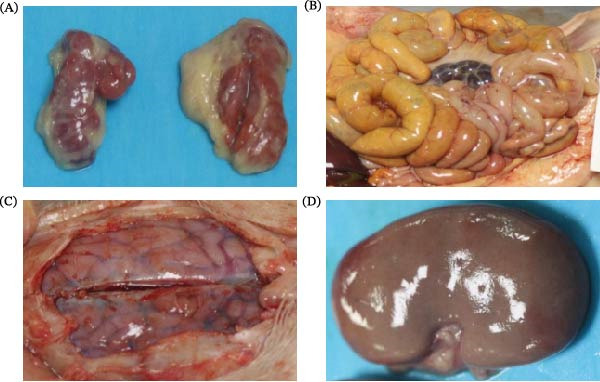
Gross pathological findings in diseased tissues. (A) Inguinal lymph nodes showing marked enlargement accompanied by extensive hemorrhage. (B) Small intestine markedly distended with abundant yellowish contents, associated with thinning of the intestinal wall and hemorrhagic changes in the mesentery. (C) Subdural hemorrhage observed in the meninges. (D) Kidneys showing multifocal petechial hemorrhages distributed throughout the renal cortex.

Gross pathological examination revealed consistent lesions in diseased piglets. Marked cyanosis of the abdominal and submandibular skin was evident. The inguinal lymph nodes were enlarged and hemorrhagic (Figure 1A). The small intestine was distended and filled with abundant yellowish contents, accompanied by hemorrhage and enlargement of the mesenteric lymph nodes (Figure 1B). Subdural hemorrhage was observed in the meninges (Figure 1C), and multifocal pinpoint hemorrhagic lesions were present in the kidneys (Figure 1D).

### 3.2. Pathogen Detection

**Figure 2:**
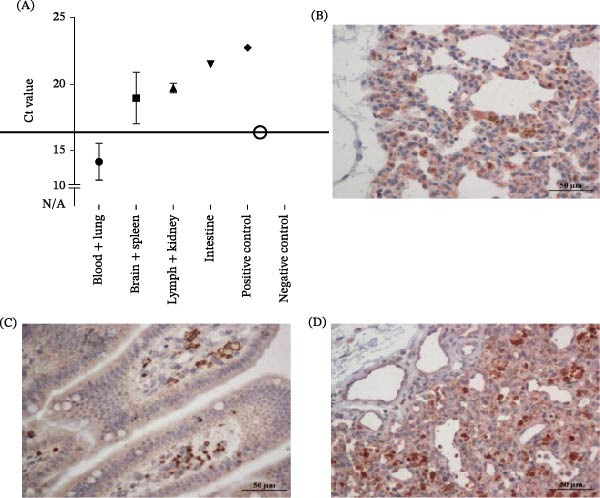
Detection and tissue distribution of Getah virus (GETV) in tissues. (A) Quantitative RT–PCR analysis showing the viral RNA loads in different tissues. (B) Immunohistochemistry (IHC) analysis of lung tissue showing strong positive staining for GETV antigens in alveolar epithelial cells and interstitial cells, accompanied by alveolar septal thickening and inflammatory cell infiltration. (C) IHC analysis of jejunal tissue demonstrating localization of GETV antigens in villous epithelial cells and the lamina propria, associated with villous disorganization and mucosal injury. (D) IHC analysis of inguinal lymph node tissue revealing widespread GETV antigen positivity in lymphoid cells, accompanied by disruption of normal architecture and hemorrhagic changes. Scale bars = 50 μm.

Screening for common swine bacterial and viral pathogens revealed that all tested bacterial agents and major enteric viruses, including PEDV, TGEV, and PoRV, were not detected in any of the examined samples. In contrast, GETV RNA was consistently detected in all collected tissues, including blood, lung, brain, lymph nodes, and intestinal samples. Ct values ranged from 12.74 to 22.81 (Figure 2A), indicating substantial viral loads across multiple organs, with particularly high levels observed in blood and lung tissues. Consistent with these molecular findings, IHC analysis demonstrated GETV antigen positivity in multiple organs, accompanied by varying degrees of tissue injury. In lung tissues, viral antigens were associated with alveolar structural disruption and interstitial inflammation (Figure 2B). In the jejunum, GETV antigens were localized to damaged villous epithelium and inflamed lamina propria (Figure 2C). In inguinal lymph nodes, extensive antigen distribution was observed in association with architectural disruption and hemorrhagic lesions (Figure 2D). Collectively, these findings indicate that GETV infection is associated with significant pathological damage in respiratory, intestinal, and lymphoid tissues.

### 3.3. Virus Isolation and Biological Characteristics

**Figure 3:**
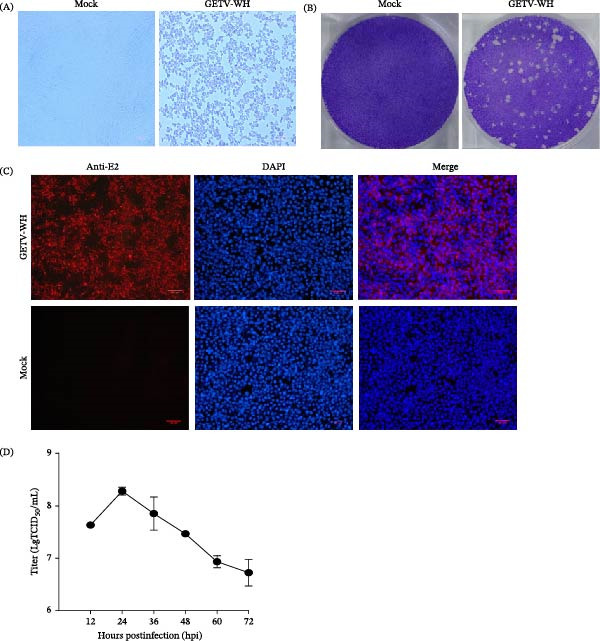
Isolation, identification, and growth characteristics of the GETV‐WH strain. (A) CPEs observed in BHK‐21 cells infected with lung tissue homogenates, characterized by progressive cell shrinkage, rounding, and detachment from the monolayer. (B) Representative plaques formed on BHK‐21 cell monolayers during plaque purification of the GETV‐WH isolate. (C) Immunofluorescence assay (IFA) showing specific fluorescence signals in BHK‐21 cells infected with the GETV‐WH isolate using a monoclonal antibody against the GETV E2 protein; no specific fluorescence was detected in mock‐infected control cells. (D) One‐step growth curve of the GETV‐WH strain in BHK‐21 cells. Viral titers increased from 12 h postinfection, reached a peak exceeding 10^8^ TCID_50_/mL at 24 h postinfection, and subsequently declined at later time points.

Following inoculation of BHK‐21 cells with lung tissue homogenates, typical and reproducible CPEs were observed, characterized by progressive cell shrinkage, rounding, and detachment from the monolayer (Figure 3A). Plaque assays revealed the formation of well‐defined plaques, and plaque purification was subsequently performed to obtain a clonal viral isolate (Figure 3B), designated GETV‐WH.

The identity of the isolated virus was further confirmed by IFA using a monoclonal antibody specific for the GETV E2 protein. Strong and specific fluorescence signals were detected in infected BHK‐21 cells, whereas no fluorescence was observed in mock‐infected control cells (Figure 3C), confirming successful isolation of GETV.

Growth kinetics analysis demonstrated that viral titers began to increase at 12 h postinfection, reached a peak at 24 h postinfection with titers exceeding 10^8^ TCID_50_/mL, and subsequently declined at later time points (Figure 3D). Taken together, these results demonstrate the successful isolation, identification, and efficient replication of GETV‐WH in BHK‐21 cells.

### 3.4. Genomic and Phylogenetic Analyses

Comparative sequence analysis revealed distinct amino acid substitution patterns in the GETV‐WH strain (GenBank accession number: PV235474), relative to the reference strain MM2021 (Figure 4A). The majority of amino acid substitutions identified in GETV‐WH occurred at high frequencies among other GETV strains, suggesting that these mutations represent common evolutionary variations within the circulating viral population. In contrast, a limited number of substitutions were detected at low frequencies or were absent in most analyzed strains, indicating potential strain‐specific genetic features. Notably, two amino acid substitutions, A476V in NSP1 and A223T in the E2 protein, were identified as unique to the GETV‐WH strain, as these were not observed in any other GETV genomes included in the analysis. These unique substitutions further highlight the distinct genetic characteristics of the GETV‐WH isolate. Phylogenetic analysis based on complete genome sequences demonstrated that all analyzed GETV strains clustered into four major genetic groups (Groups I–IV) (Figure 4B) (Table S1). The GETV‐WH strain was assigned to Group III and clustered with recent GETV isolates predominantly originating from China, indicating close genetic relatedness at the group level. Notably, GETV‐WH did not cluster within any previously reported strain‐level subclade but instead formed a distinct and independent branch within Group III, suggesting that it represents a genetically divergent lineage among the currently available GETV sequences. High bootstrap support values at major nodes confirmed the robustness of the phylogenetic topology.

Figure 4Amino acid substitution patterns and phylogenetic analysis of the GETV‐WH strain. (A) Frequency distribution of amino acid substitutions identified in the GETV‐WH strain relative to the reference strain MM2021 across 85 complete GETV genomes. Each dot represents an individual amino acid substitution. Gray dots indicate common substitutions, red dots indicate rare substitutions, and open red circles indicate substitutions unique to GETV‐WH. The dashed line represents the frequency cutoff (0.15) used to distinguish common from rare substitutions. Viral genomic regions are indicated along the *x*‐axis. (B) Phylogenetic tree constructed based on complete genome sequences of 86 GETV strains using the neighbor‐joining method with 1000 bootstrap replicates. Bootstrap values ≥ 50% are shown at the corresponding nodes. The GETV‐WH strain is highlighted in red with a star. Branch colors indicate the geographic origin of each strain, whereas background shading denotes host species. Genetic group classifications (Groups I–IV) are indicated on the right.

**Figure:**
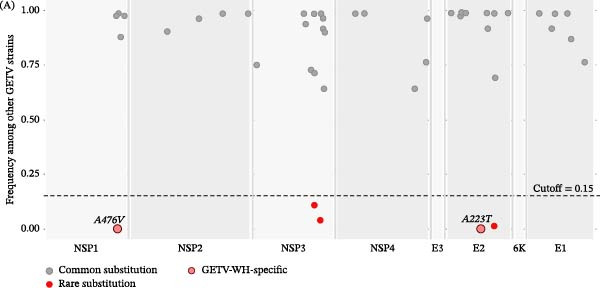

**Figure:**
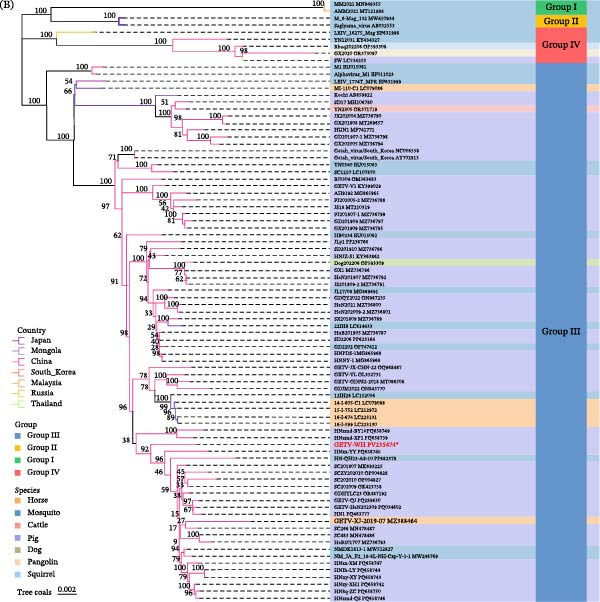

### 3.5. Pathogenicity and Tissue Distribution of GETV‐WH in Piglets

**Figure 5:**
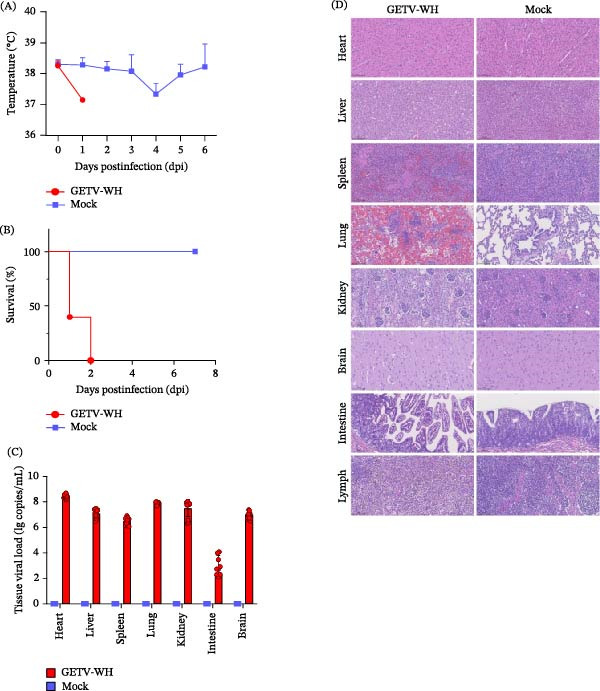
Pathogenicity and tissue distribution of the GETV‐WH strain in neonatal piglets. (A) Changes in rectal temperature of piglets following intramuscular inoculation with GETV‐WH or DMEM (mock control). Piglets infected with GETV‐WH showed a decrease in body temperature beginning at 1 day postinoculation, whereas temperatures in the mock group remained stable throughout the observation period. (B) Survival curves of GETV‐WH–infected and mock‐infected piglets. All piglets inoculated with GETV‐WH succumbed within 2 days postinoculation, while all mock‐infected piglets survived throughout the experimental period. (C) Viral RNA loads in different tissues of piglets determined by real‐time RT–PCR. High levels of GETV RNA were detected in multiple organs, including heart, liver, spleen, lung, kidney, intestine, and brain, from GETV‐WH–infected piglets, whereas no viral RNA was detected in tissues from mock‐infected controls. (D) Representative histopathological changes in tissues from GETV‐WH–infected and mock‐infected piglets stained with hematoxylin and eosin (H&E). Severe lesions were observed in the lungs, characterized by alveolar hemorrhage and inflammatory cell infiltration, along with notable pathological alterations in the intestine, lymph nodes, spleen, and kidney. Tissues from mock‐infected piglets showed normal histological architecture (bar = 100 μm).

To evaluate the pathogenicity of the isolated GETV‐WH strain in vivo, experimental infection was performed in 5‐day‐old piglets. Following intramuscular inoculation, piglets in the GETV‐WH–infected group did not exhibit fever; instead, a noticeable decrease in rectal temperature was observed beginning at 1 day postinoculation. In contrast, rectal temperatures in mock‐infected piglets remained stable throughout the observation period (Figure 5A). Survival analysis revealed marked lethality in GETV‐WH–infected piglets. All infected animals died within 2 days postinoculation, whereas all mock‐infected piglets (100%) survived until the end of the experiment (Figure 5B), indicating that the GETV‐WH strain is highly virulent in neonatal piglets. Quantitative real‐time RT–PCR analysis demonstrated systemic viral dissemination in infected piglets. High viral RNA loads were detected in multiple organs, including the heart, liver, spleen, lung, kidney, intestine, and brain, with particularly elevated levels observed in the lung, heart, and brain tissues (Figure 5C). No viral RNA was detected in tissues collected from mock‐infected animals.

Histopathological examination revealed severe tissue damage associated with the GETV‐WH infection (Figure 5D). Lung tissues from infected piglets exhibited extensive alveolar hemorrhage, inflammatory cell infiltration, and disruption of the normal alveolar architecture. Intestinal tissues showed villous damage and inflammatory lesions, while the lymph nodes displayed hemorrhage and lymphoid depletion. Mild to moderate pathological alterations were also observed in the spleen and kidney. In contrast, tissues from mock‐infected piglets maintained normal histological structures without evident lesions. Taken together, these findings indicate that GETV‐WH causes acute and lethal infection in neonatal piglets, characterized by hypothermia rather than fever, rapid mortality, systemic viral replication, and severe pathological damage in multiple organs.

## 4. Discussion

GETV is an emerging mosquito‐borne alphavirus that has garnered increasing attention due to its expanding host range and growing impact on swine health [16]. In this study, we isolated and characterized a novel GETV strain, designated GETV‐WH, which exhibits high pathogenicity in neonatal piglets under experimental conditions. Through integrated virological, pathological, and genomic analyses, our findings provide insights into the biological characteristics, virulence, and evolutionary dynamics of currently circulating GETV strains.

A notable feature of GETV‐WH infection was its rapid and lethal disease course in neonatal piglets [17], characterized by early hypothermia and death within a short time frame following inoculation. In contrast to the febrile responses commonly observed during many viral infections, including previously described GETV infections [18–20], hypothermia was observed in the present study. However, differences in experimental conditions, including the inoculation dose and age of piglets, may significantly influence disease outcomes. Previous studies have reported variable mortality rates and disease severity among different GETV strains, suggesting heterogeneity in virulence across circulating isolates. Dose‐dependent effects were not evaluated in the present study, and therefore, the classification of GETV‐WH as a highly virulent strain should be interpreted within the context of the current experimental conditions. Notably, a discrepancy in body temperature response was observed between experimental infection and field cases. While experimentally infected piglets showed hypothermia, clinically affected piglets initially presented with fever. This difference may be attributed to multiple factors, including the infection route, inoculation dose, and physiological status of the animals. High‐dose experimental inoculation may accelerate systemic deterioration, leading to hypothermia rather than a typical febrile response [12, 21]. In this study, intramuscular inoculation was used to evaluate the pathogenicity of GETV‐WH, following previously reported experimental designs [7, 20]. This approach allows for controlled and reproducible infection. However, it does not fully mimic the natural mosquito‐borne transmission route of GETV. Therefore, the observed pathogenicity should be interpreted with caution, as artificial inoculation may influence viral dissemination and disease progression.

The high lethality observed following GETV‐WH was accompanied by extensive systemic viral dissemination and severe multiorgan pathology. Viral RNA was detected at high levels across multiple organs, including the lung, heart, brain, intestine, and lymphoid tissues, consistent with widespread viral replication [22]. Histopathological analysis further revealed marked tissue damage, particularly in the respiratory and intestinal systems, as well as hemorrhagic and inflammatory lesions in lymphoid and parenchymal organs [17]. Together, the combination of uncontrolled viral spread and severe tissue injury provides a clear pathological basis for the rapid clinical deterioration and high mortality observed in infected piglets, suggesting that GETV infection can result in acute systemic disease rather than a localized infection.

Genomic analysis revealed that while most amino acid substitutions in GETV‐WH were shared with other circulating GETV strains, two substitutions, V476 in NSP1 and T223 in the E2 protein, were unique to this isolate. These substitutions represent strain‐specific genetic features of GETV‐WH. Although NSP1 is involved in viral RNA replication and E2 plays a role in viral entry and host interaction, the functional significance of these substitutions remains unclear and requires further investigation using reverse genetics approaches [23–25]. Phylogenetically, GETV‐WH clustered within Group III alongside other recently reported GETV strains, indicating that it belongs to a currently circulating genetic lineage. Notably, GETV‐WH formed an independent branch within this group, reflecting ongoing viral diversification. This genetic divergence, together with the pronounced pathogenicity observed in neonatal piglets, highlights the importance of continuous surveillance of GETV, not only in terms of prevalence but also in terms of changes in the pathogenic potential.

In conclusion, this study reports a GETV strain associated with severe disease and high mortality in neonatal piglets under experimental conditions. The findings expand current knowledge on the pathogenicity and genetic diversity of GETV and provide a basis for future studies aimed at elucidating the molecular determinants of virulence and improving disease control strategies.

## 5. Conclusion

In this study, a highly pathogenic GETV strain, designated GETV‐WH, was successfully isolated and comprehensively characterized. The isolate exhibited efficient replication and pronounced CPEs in vitro. In vivo infection experiments demonstrated that GETV‐WH caused acute and lethal diseases in neonatal piglets, characterized by hypothermia, rapid mortality, systemic viral dissemination, and pathological lesions affecting multiple organs. Tissue distribution analyses confirmed broad organ tropism, with high viral loads detected in both central and peripheral tissues, which correlated well with the observed histopathological damage. Genomic and phylogenetic analyses revealed that GETV‐WH belongs to genetic Group III but forms a distinct phylogenetic branch, reflecting ongoing genetic diversification among circulating GETV strains. Notably, unique amino acid substitutions identified in the NSP1 and E2 proteins may be associated with the biological and pathogenic characteristics of this isolate, although their precise functional roles warrant further investigation. Collectively, these findings demonstrate the emergence of a highly virulent GETV strain with strong replication capacity, broad tissue tropism, and distinct genetic features. This study provides important insights into the pathogenic potential and evolutionary dynamics of GETV and highlights the necessity for continued surveillance, molecular characterization, and pathogenicity assessment of GETV in swine populations to better inform disease prevention and control strategies.

## Funding

This work was supported by Fundamental and Interdisciplinary Disciplines Breakthrough Plan of the Ministry of Education of China (Grant JYB2025XDXM704), Self‐designed research project for Hainan Institute of Huazhong Agricultural University (Grant 2025HZAUHNZS001), the Innovation Fund of International Joint Research Center of National Animal Immunology (Grant 2025IJRCNAI09), National Key R&D Program of China (Grant 2025YFC3507700), China Agriculture Research System of MOF and MARA (Grant CARS‐35), Key Research and Development Program of Jiangxi Province (Grant 20261BCF320004) and Yingzi Tech & Huazhong Agricultural University Intelligent Research Institute of Food Health (Grant IRIFH202209).

## Ethics Statement

All animal experiments were conducted in accordance with institutional guidelines for the care and use of laboratory animals. The study protocol was reviewed and approved by the Animal Welfare and Ethics Committee of Wuhan Keqian Biology Co., Ltd. (Approval Number SOR‐AR‐022).

## Conflicts of Interest

The authors declare no conflicts of interest.

## Supporting Information

Additional supporting information can be found online in the Supporting Information section.

## Supporting information


**Supporting Information** Table S1: Contains the information of GETV strains used for phylogenetic analysis in this study, including strain names, host species, geographic origins, years of isolation, and GenBank accession numbers.

## Data Availability

The data that support the findings of this study are available from the corresponding author upon reasonable request.
